# Dual-Functional Fluorescent Probe in the Diagnosis of Liver Injury and the Evaluation of Drug Therapy with Double Signal Amplification

**DOI:** 10.1021/cbmi.3c00128

**Published:** 2024-01-26

**Authors:** Chenchen Bian, Miaomiao Liu, Jiayi Cheng, Lei Yang, Zhanxian Li, Mingming Yu

**Affiliations:** † Green Catalysis Center and College of Chemistry, Zhengzhou University, Zhengzhou, 450001, China; ‡ Shandong Provincial Key Laboratory of Detection Technology for Tumor Markers, College of Chemistry and Chemical Engineering, Linyi University, Linyi 276000, China

**Keywords:** viscosity, polarity, diabetes, liver injury, bioimaging

## Abstract

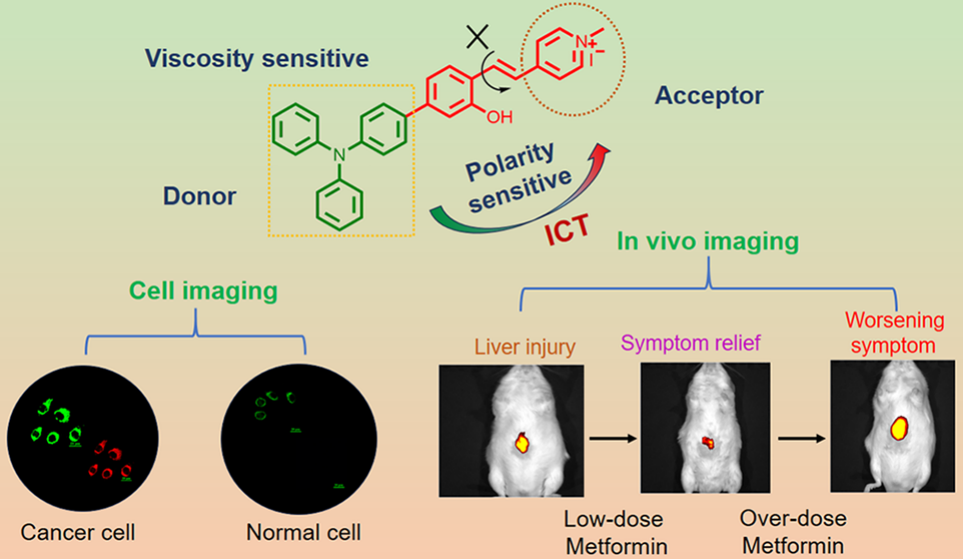

Viscosity and polarity are crucial microenvironmental parameters within cells, intimately linked to the physiological activities of organisms. We constructed and synthesized an innovative dual-functional fluorescent probe, DHBP. In the green channel, the fluorescence signal notably intensifies with decreasing environmental polarity, while in the red channel, fluorescence signal amplification occurs due to the collaborative effects of viscosity and polarity, resulting in more pronounced changes. Additionally, DHBP demonstrates high sensitivity in detecting changes in polarity and viscosity induced by drug-induced inflammation in cells and mice. Importantly, DHBP has been effectively utilized to monitor alterations in viscosity and polarity in the liver injury induced by diabetes in vivo in mice and further employed to assess the therapeutic efficacy of drugs. Therefore, DHBP holds promise for advancing research on viscosity and polarity in future studies of physiological and pathological processes.

## 1 Introduction

Diabetes, also referred to as hyperglycemia, is a chronic metabolic disorder primarily distinguished by inadequate insulin production or diminished cellular sensitivity to insulin.^1−3^ This results in the body’s inability to efficiently convert blood glucose into energy or store it.^4^ Its principal hallmark is the persistent elevation of blood glucose levels in the body, surpassing the normal range. Diabetes is a major contributor to cardiovascular diseases,^5^ amputations, blindness,^6^ and various tissue and organ damages.^7−9^ As a metabolic disease, diabetes adversely affects liver function. Without timely detection and treatment in diabetic patients, it can lead to further impairment of liver function and subsequent liver injury. Metformin, widely used to treat type 2 diabetes, operates through a mechanism that inhibits endogenous glucose production mediated by the liver. Research indicates that long-term metformin treatment in diabetes patients can lead to serious side effects,^10,11^ primarily manifested as liver injury. Therefore, monitoring the therapeutic efficacy of metformin is crucial for diabetes management and the protection of liver tissues. Developing a noninvasive, efficient method to detect and determine the extent of liver damage in diabetes is imperative.

Intracellular microenvironments, encompassing factors such as viscosity and polarity, play a crucial role in upholding redox balance and controlling the diffusion of active substances.^12,13^ Aberrations in these factors can result in cellular damage.^14^ Viscosity, a critical parameter within the cellular microenvironment,^15^ is integral to various physiological processes at the cellular level, including enzyme catalysis,^16^ signal transmission,^17^ protein aggregation,^18^ and molecular diffusion.^19,20^ Polarity, as another vital microenvironmental parameter, plays a crucial role in cell differentiation,^21,22^ proliferation, immune system regulation, and other related physiological processes.^23−25^ Anomalies in intracellular viscosity and polarity values can reduce the activity of membrane-bound proteins,^26−28^ contributing to various diseases,^29^ including fatty liver, Alzheimer’s disease, inflammation, and diabetes.^30,31^ Consequently, precise monitoring of changes in polarity and viscosity, especially within biological systems, holds paramount significance in comprehending the pathological implications of diseases, understanding physiological processes, and enabling early diagnosis.^32−34^

The emergence of diabetes is intricately linked to abnormalities in microenvironmental factors such as viscosity and polarity within the body.^35^ Consequently, the development of a highly sensitive, noninvasive imaging tool to explore the intricate relationship between viscosity, polarity, and diabetes is of paramount significance.^36,37^ Presently, the predominant approach for diabetes diagnosis hinges on blood glucose level measurements and the application of diverse assay kits for the detection of disease-related biomarkers. In certain instances, the condition only becomes discernible when patients manifest evident complications, frequently resulting in the missed opportunity for an optimal diagnosis. Additionally, conventional diagnostic methods still have limitations, such as low specificity,^38,39^ cumbersome processes, and notable risks.^40,41^ Recently, fluorescence optical imaging and analysis techniques have played a pivotal role in early disease diagnosis, treatment, new drug development, and drug delivery,^42,43^ owing to noninvasive nature,^44^ high sensitivity, and simple operation.^45−48^ Hence, the development of an appropriate fluorescence probe to elucidate the mechanisms, classification, and the biological and pathological functions related to diabetes and associated diseases with viscosity and polarity holds profound significance.^49,50^

**Scheme 1:**
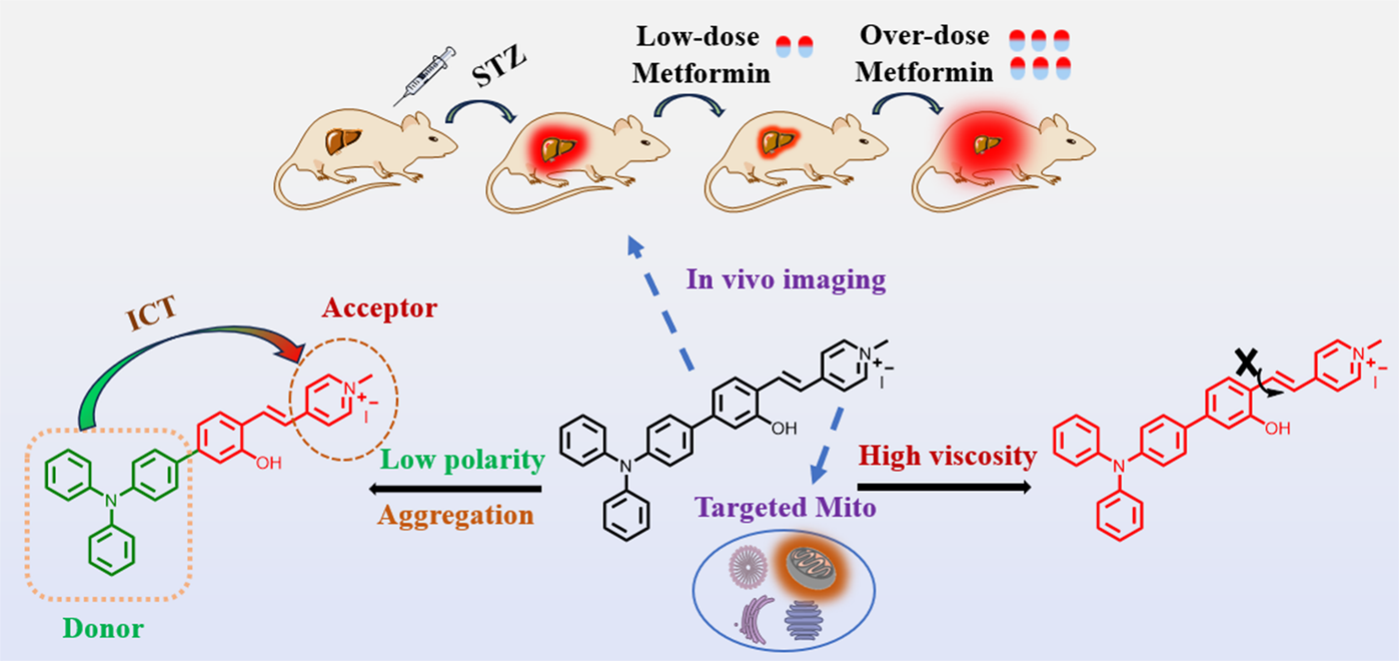
Mechanism of DHBP for Viscosity and Polarity Detection

In this study, an innovative dual-functional fluorescent probe capable of simultaneously detecting viscosity and polarity was designed and developed. The probe (*E*)-4-(2-(4′-(diphenylamino)-3-hydroxy-[1,1′-biphenyl]-4-yl) vinyl)-1-methylpyridin-1-ium iodide (DHBP) is constructed by combining a triphenylamine derivative and an electron-deficient tetramethylpyridine derivative through a vinyl linkage. In this structural arrangement, the triphenylamine component serves as an electron donor, while the positively charged tetramethylpyridine segment functions as an electron acceptor, resulting in the formation of a typical “D-π-A” architecture (Scheme 1). This configuration displays exceptional sensitivity to polarity, with the triphenylamine moiety imparting the probe with noteworthy aggregation-induced emission (AIE) characteristics.^51^

Hence, DHBP possesses several advantages: (i) DHBP enables dual-channel simultaneous detection of viscosity and polarity. (ii) DHBP exhibits remarkable selectivity for viscosity and polarity both in vitro and in vivo. (iii) Viscosity and polarity contribute to signal superposition in the red channel, resulting in signal amplification and enhanced sensitivity. (iv) DHBP has been successfully employed for monitoring changes in polarity and viscosity in mice with inflammation and liver injury caused by diabetes, and it can be used to evaluate the effectiveness of drug treatments.

## 2 Experimental Section

### 2.1 Materials and Apparatus

4-Methylpyridine, iodomethane, acetonitrile, diethyl ether, 4-boronic acid triphenylene, 4-bromo-2-hydroxybenzaldehyde, THF, K_2_CO_3_, tetrabutylammonium bromide, tetrakis(triphenylphosphine)palladium, and EtOH were used. The reagents mentioned above are of analytical grade and can be used directly. The water used for the experiment is double steamed water. The instruments and biological experimental methods used during the experimental process are included in the Supporting Information.

### 2.2 Synthesis of A1and A2

The synthesis routes and characterization results of intermediate products A1 and A2 are presented in the Supporting Information of this paper, while the detection mechanism of DHBP can be found in Scheme 1.

### 2.3 Synthesis of DHBP

A mixture of A1 (94 mg, 0.57 mmol) and A2 (125 mg, 0.34 mmol) was placed in a round-bottom flask. Approximately 7 mL of ethanol was added, and the mixture was refluxed overnight at 78 °C. The system was brought to room temperature, followed by filtration and washing with an ether solution to obtain brown-red DHBP (215 mg, yield: 37%). ^1^H NMR (600 MHz, DMSO-*d*_6_) δ (ppm) 8.77 (d, *J* = 6.6 Hz, 2H), 8.17 (d, *J* = 6.6 Hz, 2H), 8.09 (d, 1H), 7.72 (d, 1H), 7.58 (t, *J* = 8.6 Hz, 3H), 7.34 (t, 4H), 7.19 (s, 2H), 7.04 (d, *J* = 8.6 Hz, 8H), 4.23 (s, 3H). ^13^C NMR (151 MHz, DMSO-*d*_6_) δ 158.6, 153.7, 147.8, 147.4, 145.2, 143.3, 136.9, 133.2, 130.1, 130.0, 127.9, 124.9, 123.9, 123.5, 123.3, 122.6, 121.3, 117.7, 114.1, 47.1. (Figures S15 and S16) HRMS (EI) *m*/*z*: calcd for C_32_H_27_N_2_O^+^[M – I]^+^, 455.2157; found, 455.2123 (Figure S17).

### 2.4 Preparation of Solutions

The probe stock solution used in the testing process was prepared by diluting DMSO of chromatographic purity. Various interference ions were prepared using distilled deionized water. (MgCl_2_, KCl, FeCl_2_, CaCl_2_, CuCl_2_, ZnCl_2_, MnCl_2_, NaCl, NaF, NaBr, Na_2_S, NaClO, Na_2_CO_3_, NaHSO_3_, H_2_O_2_, Cys, GSH). To assess the probe’s polarity characteristics (AIE property), testing systems comprising varying ratios of CHCl_3_/DMSO were chosen. To investigate the probe’s viscosity properties, testing systems incorporating different ratios of glycerol and PBS buffer solution were selected.

## 3 Results and Discussion

### 3.1 Design of DHBP

For the design of fluorescent probe DHBP based on AIE characterization, A typical “D-π-A″ structure is constructed with a cationic tetramethylpyridine segment as the electron acceptor and a triphenylamine structure as the electron donor, allowing the probe to exhibit good polarity-sensitive properties. The exceptional sensitivity of DHBP to viscosity can be explained by its ability to counteract the TICT (twisted intramolecular charge transfer) effect in high-viscosity conditions. This leads to a reduction in nonradiative attenuation and, consequently, an enhancement in fluorescence. Furthermore, the cationic structure within the probe may possess targeting capabilities toward mitochondria^52,53^ (Scheme 1).

### 3.2 Spectral Response of DHBP to Polarity and AIE Features

Initially, the photophysical properties of DHBP in various polar organic solvents were examined through ultraviolet–visible absorption spectroscopy and fluorescence emission spectroscopy. As shown in Figure S1A, in solvents with higher polarity, the probe exhibits a maximum absorption wavelength concentrated around 405 nm, while in solvents with lower polarity, the probe’s maximum absorption wavelength is near 500 nm. These findings suggest a significant alteration in the dipole moment of the probe’s ground state as the solvent polarity changes. As depicted in Figure S1B and S1C, stronger fluorescence is observed in low-polarity solvents such as DCM and CHCl_3_, with variations in the emission peak positions. These findings suggest that the dipole moment of DHBP undergoes significant changes in different solvents when in the excited state, highlighting its sensitivity to polarity. Concurrently, it came to our attention that the probe demonstrates significant fluorescence when in glycerol, leading us to hypothesize that it may function as a viscosity-sensitive probe.

**Figure 1:**
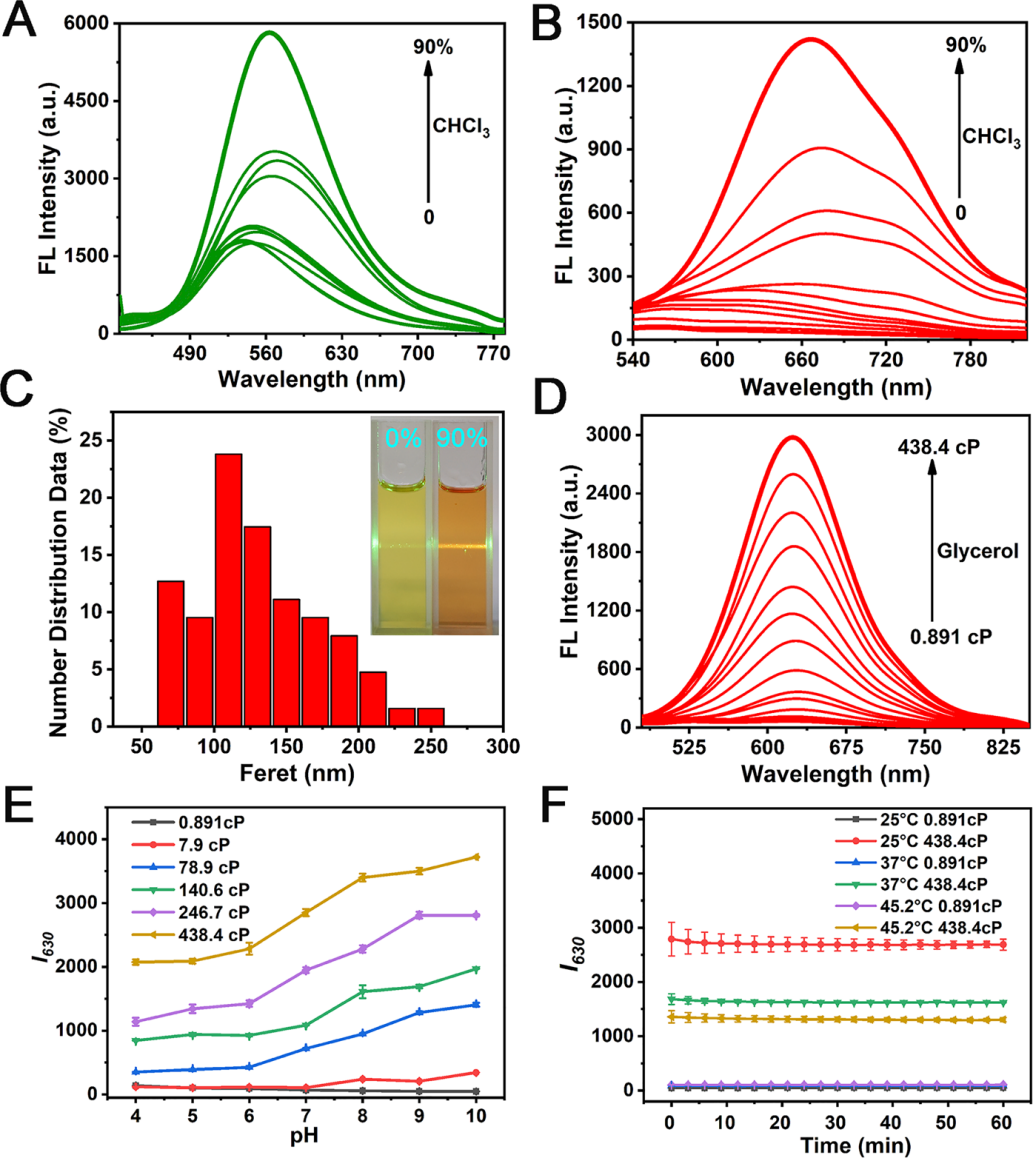
Fluorescence emission spectra of DHBP (1.0 × 10^–5^ mol/L) in varying volumes of CHCl_3_/DMSO solution. (A, λ_ex_ = 405 nm; B, λ_ex_ = 480 nm, slits: 10 nm, 10 nm). (C) Particle size distribution of DHBP dissolved in 90% CHCl_3_ obtained by TEM. Insert: photos of Tyndall effect of DHBP under a laser pointer. (D) Fluorescence emission spectra of DHBP (1.0 × 10^–5^ mol/L) in PBS (pH = 7.3)–glycerol mixture systems with different ratios. (λ_ex_ = 450 nm, slits: 10 nm, 10 nm). (E) Fluorescence intensity of DHBP (1.0 × 10^–5^ mol/L) at 630 nm in solutions with different viscosity and pH. (F) Photostability of DHBP (1.0 × 10^–5^ mol/L) in mixed solutions with varying viscosity values at different temperatures, λ_ex_ = 450 nm, slits: 10 nm, 10 nm.

To further investigate the response of DHBP to polarity and determine if it exhibits aggregation-induced emission (AIE) characteristics, the UV–vis absorption spectra and fluorescence emission spectra of DHBP were examined in solutions with varying proportions of DMSO and CHCl_3_. Figure S2 displays the normalized UV–vis spectra of the probe in different ratios of DMSO and CHCl_3_ solutions. Notably, as solvent polarity decreases, the probe exhibits a significant redshift in its maximum absorption wavelength, shifting by 50 nm. When excited at 405 and 480 nm respectively, it is apparent that the fluorescence of the probe significantly is enhanced with decreasing solvent polarity. This could be attributed to DHBP displaying fewer charge separations and weaker interactions with the solvent in low-polarity media, resulting in intense fluorescence emission. However, in high-polarity media, enhanced dipole–dipole interactions between the probe and the solvent lead to reduced fluorescence emission. Figure S3 delineates the variation in fluorescence intensity emitted by the probe at 630 nm in response to alterations in the volume of CHCl_3_, demonstrating a strong linear correlation. These findings suggest that the probe has the potential to be an effective polarity-sensitive probe. Interestingly, we observed aggregation-induced fluorescence enhancement in the mixed system of DMSO (high solubility) and CHCl_3_ (low solubility) when the volume ratio of CHCl_3_ reached 90%. This phenomenon arises because in low-solubility solvents the probe tends to aggregate due to restricted molecular motion, resulting in stable and bright emission. Additionally, under laser illumination, 10 μmol/L DHBP in DMSO and 90% CHCl_3_ (with 10% DMSO) exhibited the Tyndall effect, with a more pronounced effect observed in 90% CHCl_3_ (with 10% DMSO) (insert in Figure 1C). Furthermore, transmission electron microscopy (TEM) measurements (Figure 1C) revealed that the average particle size of DHBP in 90% CHCl_3_ (with 10% DMSO) was significantly larger than that in DMSO, confirming the aggregation of DHBP in 90% CHCl_3_ (with 10% DMSO) (Figure S4). These findings suggest that DHBP exhibits typical AIE behavior in nonpolar solvents, making it a promising candidate for in vivo bioimaging applications.

### 3.3 Optical Response of DHBP to Viscosity

UV–vis absorption spectra and fluorescence emission spectra of DHBP in PBS–glycerol mixtures of different viscosities were recorded to evaluate the viscosity sensitivity of DHBP. According to Figure S5, as the proportion of glycerol increases (from 0% glycerol to 95% glycerol), the absorption peak at 450 nm gradually rises. As illustrated in Figure 1D, as the solvent viscosity increases from 0% glycerol to 95% glycerol, the fluorescence intensity of DHBP at 630 nm gradually increases. In the 95% glycerol system, the fluorescence intensity is approximately 150 times higher than that in pure PBS, and the fluorescence quantum yield increases from 0.2 to 1.2. Furthermore, as depicted in Figure S6, a strong linear correlation was observed between the solution viscosity (log η) and the fluorescence intensity (log *I*_630_) of DHBP (*R*^2^ = 0.9970) within the range of 7.9 cP to 246.7 cP. In low-viscosity media, the rotatable single bonds in DHBP induce a nonplanar molecular structure, leading to the dissipation of excited-state energy primarily through nonradiative pathways, ultimately resulting in fluorescence quenching. In contrast, the intramolecular rotation of DHBP is severely restricted in high-viscosity environments, causing a significant attenuation of excited-state energy through radiative pathways, thereby enhancing fluorescence markedly.^54^ Hence, these findings validate DHBP as a promising instrument for detecting viscosity.

Subsequently, the influence of pH on DHBP was investigated (Figure 1E). As observed, the fluorescence intensity of DHBP rises with increasing pH, assuming constant viscosity. This phenomenon can be attributed to the protonation of phenolic hydroxyl groups within the structure. Moreover, in the pH range of 4–10, the fluorescence intensity of the probe in the solution with high viscosity is always stronger than that of the solution with low viscosity value, which indicates the feasibility of detecting viscosity in this range.

Temperature plays a crucial role in influencing viscosity, making it essential to investigate its impact on the detection of viscosity with DHBP. As can be seen in Figure 1F, when the probe is in a pure PBS buffer solution, the influence of temperature on fluorescence intensity can be negligible. Conversely, in a 95% glycerol system, there is a negative correlation between temperature and fluorescence intensity, possibly due to changes in the fluidity of the liquid with temperature variations. Of greater significance, when exposed to 60 min of fluorescence irradiation, DHBP exhibits remarkable photostability.

From above research, it can be seen that DHBP has excellent viscosity response characteristics. Taking into account the practical application of the probe, the subsequent exploration focuses on the probe’s resistance to interference from various substances within the biological system. As shown in Figure S7, the fluorescence intensity of DHBP exhibits negligible changes upon the addition of various active substances. This observation indicates that DHBP has good anti-interference and specificity for viscosity in complex biological systems. (*I*_0_ represents the fluorescence intensity of DHBP at 630 nm in a PBS buffer solution, and *I* represents the fluorescence intensity subsequent to the introduction of various species.)

### 3.4 Intracellular Fluorescence Imaging of DHBP

First, the cytotoxicity of the DHBP probe was assessed using the CCK-8 assay. After co-incubation with various concentrations of DHBP for 24 h, the cell viability remained above 85%. (Figure S8) The highest concentration of the probe used was 40 μM, indicating that the probe has low toxicity within the cells.

Subsequently, as depicted in Figure S9, there was no significant alteration observed in the fluorescence imaging of DHBP within HeLa cells during the first 30 min. This observation suggests that the probe exhibits excellent stability within cellular environments. Following pretreatment with DHBP, HeLa cells were subsequently incubated with a commercial dye (Mito-Tracker Red, 1 μM). Colocalization analysis results (Figure S10) indicated substantial overlap between the red fluorescence and green fluorescence emitted by DHBP with the blue fluorescence from the commercial dye, which was well evidenced by Pearson’s colocalization coefficients of 0.94 and 0.85. This outcome strongly suggests that DHBP primarily localizes within the mitochondria. This localization can be attributed to the presence of cationic groups in DHBP, enabling the probe to target the mitochondrial inner membrane with a negative membrane potential.

**Figure 2:**
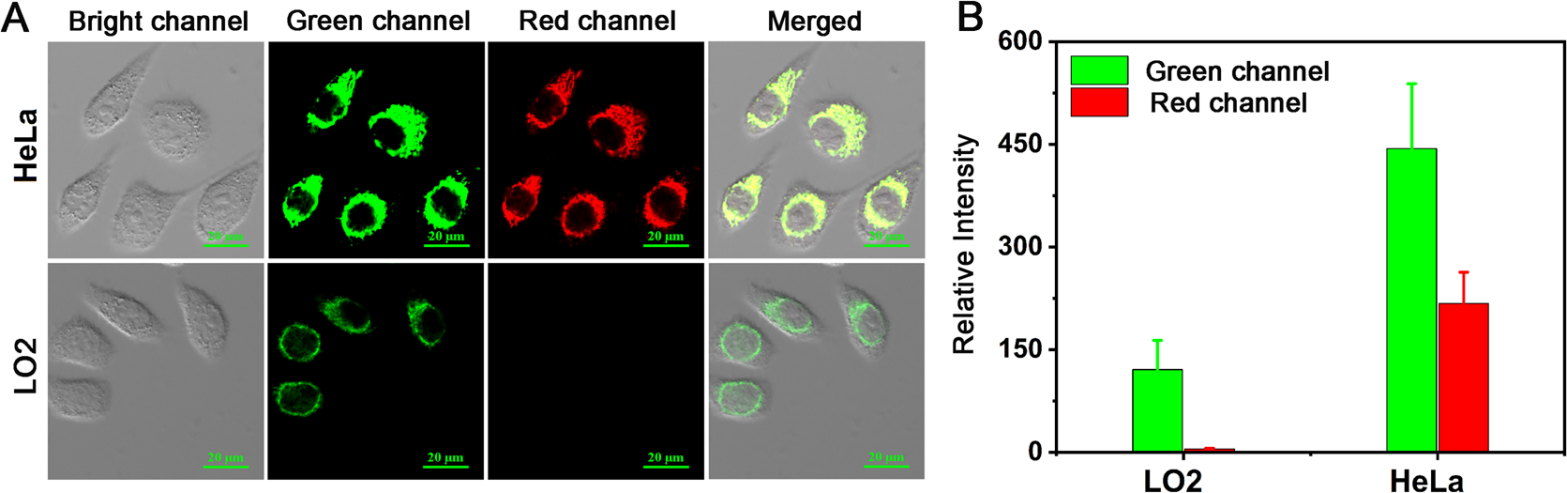
(A) Fluorescence confocal images of DHBP (20 μM) following incubation with both HeLa cells and LO2 cells. (B) Histogram of the fluorescence intensities in panel A (green channel: λ_ex_ = 405 nm, λ_em_ = 500–550 nm; red channel: λ_ex_ = 488 nm, λ_em_ = 651–720 nm).

Due to the higher viscosity and lower polarity of cancer cells compared to normal cells,^55^ we explored whether DHBP could be used to distinguish between cancer and normal cells. After co-incubating DHBP with both HeLa cancer cells and normal cells, it was observed that the fluorescence intensity of the probe exhibited distinct distributions in the two cell types (Figure 2A and 2B). Therefore, this suggests that DHBP can differentiate cancer cells from normal cells based on differences in viscosity and polarity.

**Figure 3:**
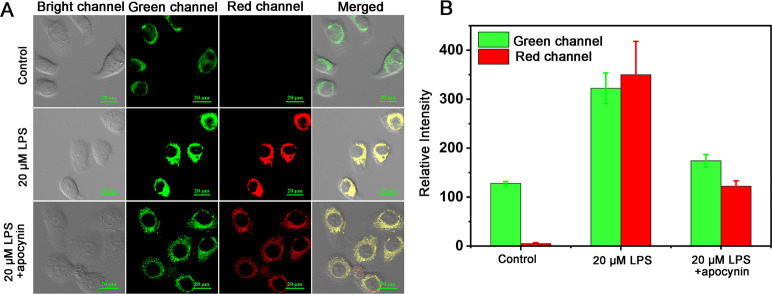
(A) Fluorescence images of 20 μM DHBP in HeLa cells pretreated with 20 μM LPS and 20 μM LPS + apocynin. (B) Histogram of the fluorescence intensities in panel A (green channel: λ_ex_ = 405 nm, λ_em_ = 500–550 nm; red channel: λ_ex_ = 488 nm, λ_em_ = 651–720 nm).

Subsequently, to investigate DHBP’s ability to monitor viscosity and polarity within cells, a drug-induced inflammation cell model was constructed to induce changes in viscosity and polarity. Lipopolysaccharide (LPS) is commonly used as an inducer for acute inflammation,^31^ which can alter the viscosity and polarity of living cells by affecting their normal functioning. Therefore, HeLa cells were cultured with a high concentration of LPS (20 μM) for 4 h, followed by the addition of DHBP for further incubation. As shown in Figure 3A and 3B, upon the addition of LPS, the fluorescence intensity in the green channel (λ_ex_ = 405 nm, λ_em_ = 500–550 nm) significantly increased, and the fluorescence intensity in the red channel (λ_ex_ = 488 nm, λ_em_ = 651–720 nm) also exhibited a substantial increase, with fluorescence intensity increasing by approximately 3-fold. Apocynin is an anti-inflammatory agent used to attenuate inflammation. After co-incubation of cells with LPS and apocynin, followed by the addition of DHBP for further incubation, the experimental results in Figure 3 demonstrate a decrease in fluorescence intensity in both the green and red channels. This suggests an alleviation of the inflammatory conditions within the cells. The changes in fluorescence emission align with the results obtained from solution tests, indicating that the probe can be used for sensitive monitoring of the significant increase in intracellular viscosity and decrease in polarity induced by drug stimulation.

### 3.5 Fluorescence Imaging of DHBP in Inflammatory Mice

**Figure 4:**
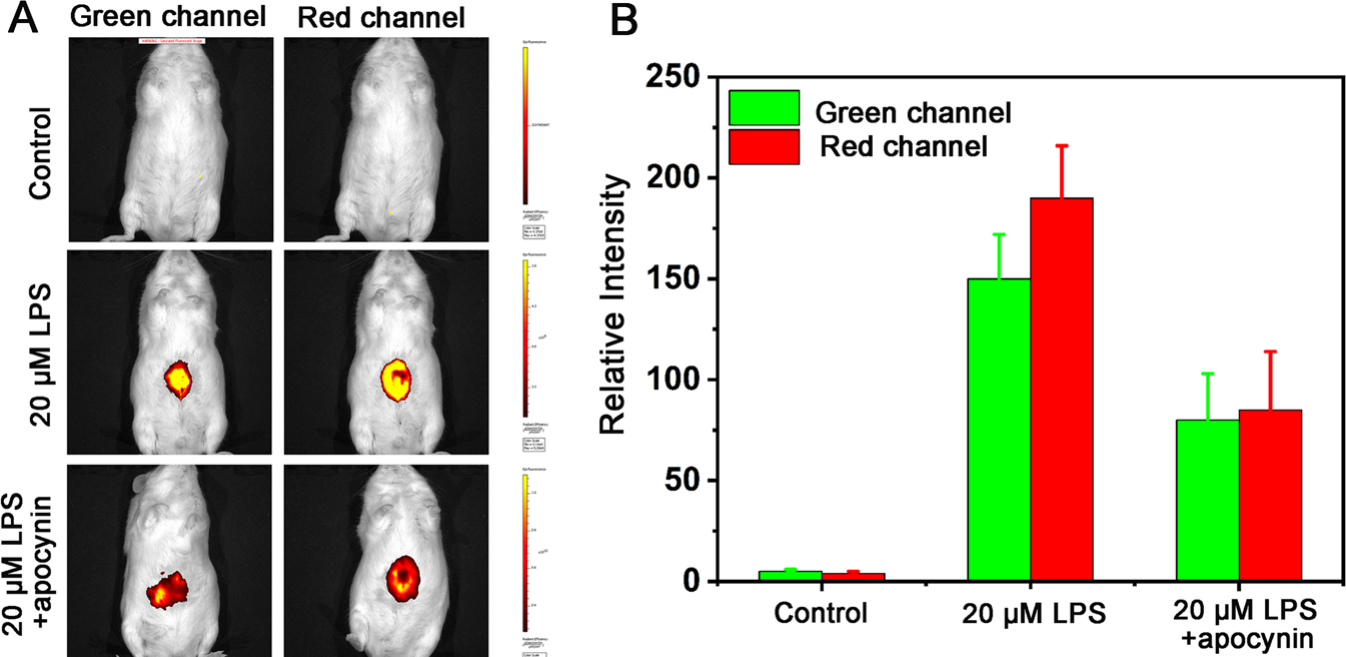
(A) In vivo imaging of DHBP in mice after different stimulations: mice treated with the probe only, mice pretreated with LPS, mice pretreated with LPS and further treated with apocynin. (B) Histogram of the fluorescence intensities in panel A (green channel: λ_ex_ = 405 nm, λ_em_ = 500–550 nm; red channel: λ_ex_ = 488 nm, λ_em_ = 651–720 nm).

Next, the effect of LPS-induced acute inflammation in mice was further investigated. The mice were randomly allocated into three groups: the first group acted as the control group and did not undergo any drug treatment, with mice receiving a 100 μL saline injection into the peritoneal cavity. The second group of mice was injected with 20 μg/mL LPS (100 μL saline), while the third group of mice was injected with 20 μg/mL LPS (100 μL saline) and 20 μg/mL apocynin. With 4 h elapsed, all groups were intraperitoneally injected with 20 μM DHBP (100 μL, DMSO/saline volume ratio = 1:9), and fluorescence imaging was conducted 30 min afterward. In accordance with Figure 4, both the green and red channels exhibited significant enhancements. Co-incubation with apocynin resulted in a reduction in fluorescence in both channels, consistent with the cell-based experimental results, Furthermore, under the synergistic influence of polarity and viscosity, the fluorescence signal in the red channel exhibited even greater sensitivity to changes. Thus, it has been confirmed that LPS can induce acute inflammation in mice, resulting in elevated viscosity and reduced polarity. DHBP can be served as an effective tool for monitoring acute inflammation and evaluating the therapeutic effects of apocynin.

### 3.6 Fluorescence Imaging of DHBP in Diabetic Mice

**Figure 5:**
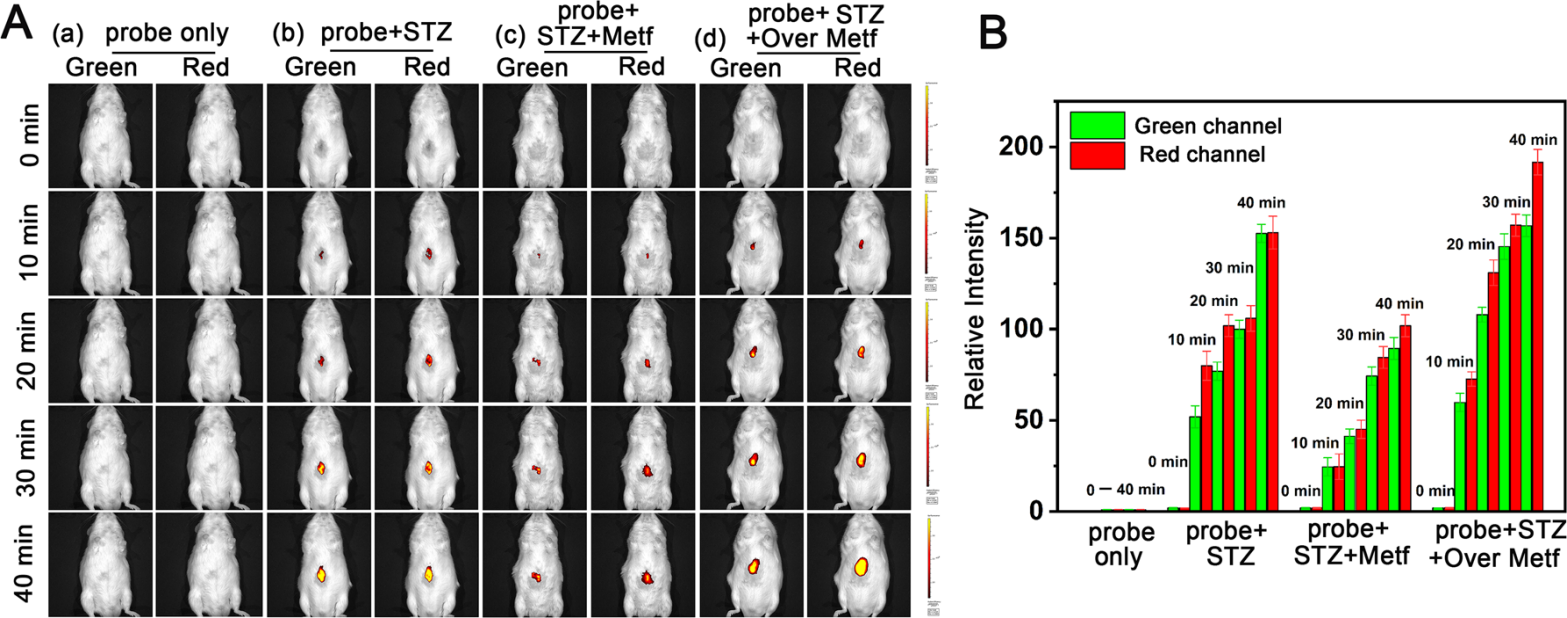
(A) Fluorescence imaging of mice in different groups within 40 min. Group a: Normal mice injected with probe only; group b: diabetic mice; group c: diabetic mice fed low-dose metformin; group (d): diabetic mice fed high-dose metformin. (B) Histogram of the fluorescence intensity in panel A) (green channel: λ_ex_ = 405 nm, λ_em_ = 500–550 nm; red channel: λ_ex_ = 488 nm, λ_em_ = 651–720 nm).

Diabetes is a metabolic disorder that affects carbohydrate, protein, and fat metabolism. It arises due to insufficient insulin secretion or impaired insulin utilization, resulting in elevated blood sugar levels as the primary symptom. This condition can potentially lead to complications such as hepatitis and liver injury. Metformin is a widely used medication in the clinical management of type 2 diabetes. However, research has indicated that prolonged or excessive use of metformin can exacerbate liver damage. Inspired by the promising capabilities of cell imaging, we conducted a study to investigate DHBP’s ability to monitor viscosity and polarity in mice with liver injury, while also assessing the therapeutic effectiveness of metformin. Streptozotocin (STZ) can induce diabetes in mice by disrupting the normal functioning of pancreatic cells and triggering an abnormal inflammatory response that leads to liver injury.^56^ Therefore, a diabetes model was established using STZ. The diabetic mice in the model were randomly divided into three groups: Group a consisted of healthy mice that did not undergo any drug treatment, Group b received a daily intraperitoneal injection of 200 μL of PBS buffer for 7 days (10 mM, pH 7.4), Group c was administered 80 mg/kg body weight oral metformin, Group d was administered 180 mg/kg body weight oral metformin. The imaging results, as depicted in Figure 5A and 5B, revealed that the control group mice exhibited only a faint fluorescence signal at the liver site even after 40 min following probe injection. This observation can reasonably be attributed to the normal viscosity and polarity values in the control group (group a) mice. In stark contrast, mice treated with STZ showed a noticeable fluorescence signal in their livers as early as 10 min after probe injection, and the fluorescence intensity increased noticeable over time. To be more specific, the fluorescence signal in the livers of the STZ-induced group was approximately 2–3 times stronger than that in group a after 40 min, confirming an increase in vivo viscosity and a decrease in polarity following STZ-induced diabetes in mice.

Moreover, the fluorescence signal in the low-dose Metf-treated group was weaker compared to group b, while the high-dose metformin group d exhibited enhanced fluorescence. This suggests that metformin can mitigate liver damage induced by diabetes to some extent, but excessive dosage can exacerbate liver injury.

**Figure 6:**
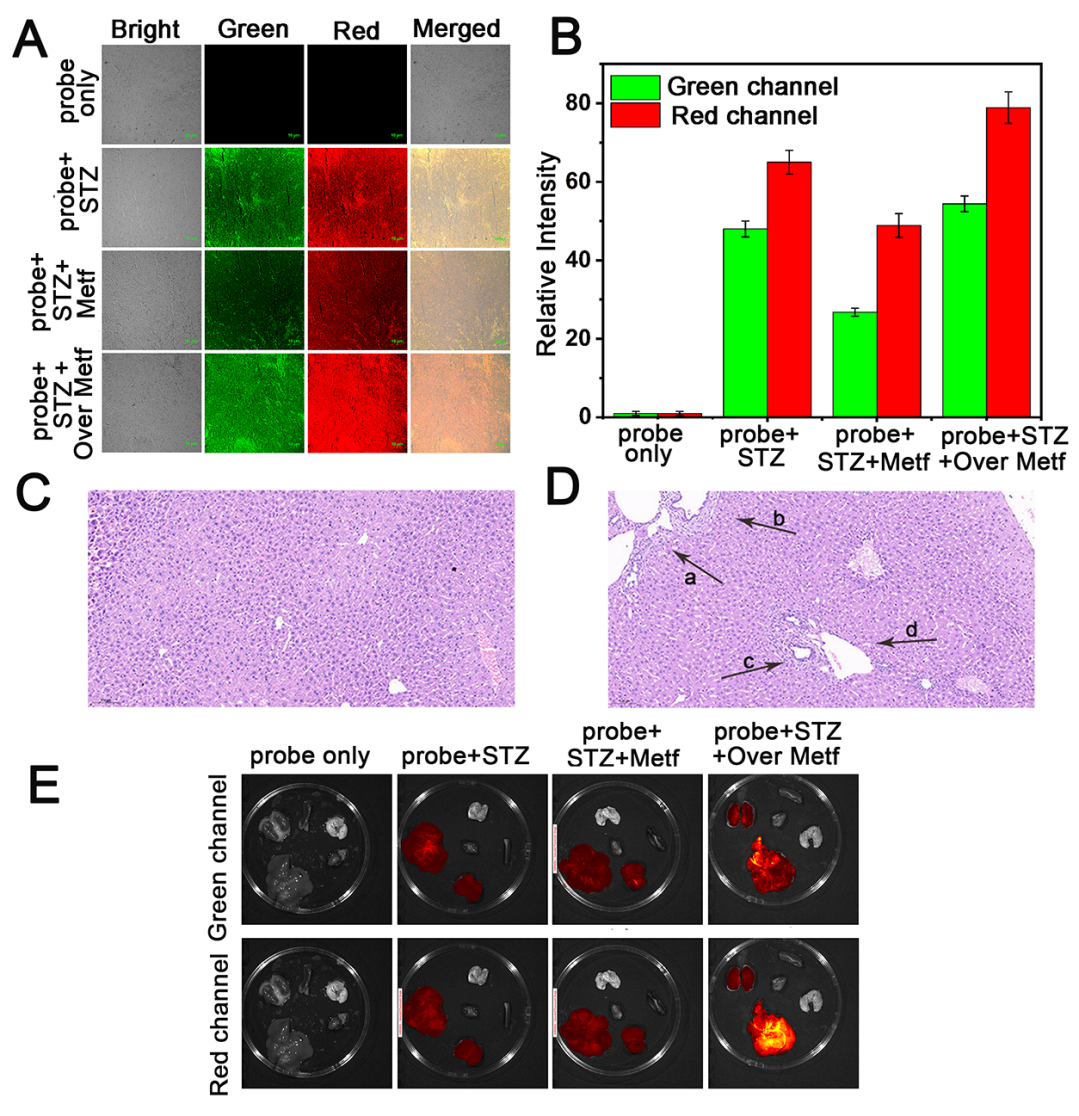
(A) Confocal image of the liver tissue of the above experimental mice. (B) Relative fluorescence intensity in (A). H&E staining images of liver tissues: (C) probe only, (D) probe + STZ. (E) Fluorescence imaging of the primary organs in the aforementioned model mice (liver, spleen, heart, lung, kidneys) (green channel: λ_ex_ = 405 nm, λ_em_ = 500–550 nm; red channel: λ_ex_ = 488 nm, λ_em_ = 651–720 nm).

Subsequently, we further examined the liver tissue by fluorescence imaging of the four groups of mice as mentioned above, and the fluorescence slice results were in harmony with in vivo imaging results (Figure 6A and 6B). The experimental findings indicate that the liver tissue of STZ-induced diabetic mice exhibits strong fluorescence signals, confirming higher viscosity and lower polarity in the liver tissue of the liver injury group compared to normal liver tissue. Symptoms of liver damage were alleviated after low-dose metformin treatment, but worsened with excessive treatment. DHBP can sensitively monitor this process through fluorescence imaging.

Furthermore, to validate whether the established mouse liver injury model aligns with expectations, pathological morphology analysis was conducted. As shown in Figure 6C and 6D, in the control group that received only the probe injection, liver tissue structures remained intact, with liver cells orderly arranged around the central vein. However, following STZ treatment, there was disrupted liver lobule arrangement and the emergence of typical hepatocellular necrosis and inflammatory infiltration in the liver organ (Figure 6D, arrows a–d). Subsequently, we conducted imaging of the major organs (liver, spleen, heart, lungs, and kidneys) of the aforementioned experimental mice, and the results are shown in Figure 6E. The fluorescence signals on the organs of the control group mice were negligible. In the STZ-induced group, fluorescence was observed in the liver and kidney organs, which may be related to the fact that the liver and kidneys are the major metabolic organs in the body. After treatment with a low dose of metformin, we observed a reduction in fluorescence signals, indicating an alleviation of the disease symptoms. However, following treatment with a high dose of metformin, a significant increase in fluorescence signals was observed in the liver organ, suggesting a further exacerbation of liver damage. In general, in vivo imaging experiments demonstrate that DHBP can effectively assess the extent of liver damage in diabetic mice and evaluate the therapeutic effects of drugs through the visualization of alterations in liver viscosity and polarity. More importantly, we utilize two biomarkers as triggers, which can improve diagnostic specificity and accuracy.^57^

## 4 Conclusion

In conclusion, we have successfully developed a novel bifunctional fluorescent probe DHBP, capable of dual detection of viscosity and polarity. Remarkably, this probe exhibits the ability to distinguish normal cells from cancer cells. Furthermore, it can effectively detect alterations in the cellular microenvironment resulting from drug–lipopolysaccharide stimulation, and has been successfully employed in mouse models. Moreover, owing to its exceptional sensitivity, DHBP enables the observation of viscosity and polarity variations, as well as the effects of drug treatments following liver injury in diabetic mice. Consequently, DHBP holds significant promise for practical applications in the study of diabetic liver injuries.

## Supporting Information Available

General information, methods, synthesis procedure, supporting figures, and characterizations (PDF)
