# Supplementary material for: Dual-Functional Fluorescent Probe in the Diagnosis of Liver Injury and the Evaluation of Drug Therapy with Double Signal Amplification
Source: Chem Biomed Imaging. 2024 Jan 26;2(2):156–64. doi: 10.1021/cbmi.3c00128 (PMC11504577; doi:10.1021/cbmi.3c00128)

Supporting information

Dual-functional Fluorescent Probe in the Diagnosis of Liver Injury and the Evaluation of Drug Therapy with Double Signal Amplification

Chenchen Bian[a], Miaomiao Liu[b], Jiayi Cheng[b], Lei Yang[b,*], Zhanxian Li[a,*] , Mingming Yu[a,*]

[a] Green Catalysis Center and College of Chemistry, Zhengzhou University, Zhengzhou, 450001, China. E-mail: yumm@zzu.edu.cn, lizx@zzu.edu.cn.

[b] Shandong Provincial Key Laboratory of Detection Technology for Tumor Markers, College of Chemistry and Chemical Engineering, Linyi University, Linyi 276000, China. Email: yanglei@lyu.edu.cn.

Contents:

1. Materials and Instruments..3

2. Experimental Section..4

2.1 Synthesis of A1and A2 4

2.2 Cytotoxicity assay and cell imaging..5

2.3 Establishment of mice model 7

2.4 Supplementary figures..8

1. Materials and Instruments

600 M nuclear magnetic resonance spectrometer (AVIII HD 600), high resolution mass spectrometer (IonSpec4.7), dual beam UV-visible spectrophotometer (TU- 1901), fluorescence spectrophotometer (F-4600), lightning magnetic pH meter (PHS-2F), constant temperature magnetic agitators (85-2), Ultrasonic cleaning machine (SB-100D), circulating water vacuum pump (SHB-3), vacuum oil pump (2XZ-4), rotary evaporation instrument (RE-2000B), electronic analytical balance (FA2004), vacuum drying oven (DZF-6020), digital camera (D3300), portable ultraviolet analyzer (ZF-5), ultra high resolution confocal microscope (Leica SP8), IVIS Lumina LT vivo imaging system.

2. Experimental Section

2.1 Synthesis of A1and A2

Synthesis of compound A1: 4-Methylpyridine (194.0 μL, 2.0 mmol) and iodomethane (6.0 mL, 96.0 mmol) were dissolved in 5 mL of acetonitrile. The mixture was heated to 45 ℃ and further refluxed for 14 h. The reaction system was cooled to room temperature, 30 mL of ether was added, and then filtered to obtain white compound A1 (250.3 mg, 53%). The structure of compound A1was verified by ^{1}H NMR and ^{13}C NMR spectra (Figures S11 and S12). ^{1}H NMR (600 MHz, $CDCl_3$) δ (ppm) 9.15 (d, *J* = 6.5 Hz, 2H), 7.89 (d, *J* = 6.3 Hz, 2H), 4.63 (s, 3H), 2.69 (s, 3H). ^{13}C NMR (151 MHz, $CDCl_3$) δ 158.9, 144.8, 128.8, 48.8, 22.5.

Synthesis of compound A2: 4-Borotriphenylacetic acid (346.8 mg, 1.2 mmol) and 4-bromo-2-hydroxybenzaldehyde (201.2 mg, 1 mmol) were dissolved in 6 mL of THF, 1.6 mL of aqueous potassium carbonate (2.0 mol/L) was added, and tetrabutylammonium bromide (262.2 mg, 0.5 mmol) was added, and the solution was stirred at room temperature for 30 min, and tetrakis (triphenylphosphine) palladium (2.4 mg, 0.002 mmol) was added to the above solution. The samples were refluxed at 68 ℃ for 7 hours under argon protection. Cooled to room temperature, the organic phase was extracted with DCM and washed with saturated salt water, spun dry and purified by column chromatography (eluent was V_{PE}: V_{EA} = 2:1) to obtain compound A2 (160.2 mg, 43.8%). The structure of compound A2 was verified by ^{1}H NMR and ^{13}C NMR spectra (Figures S13 and S14). ^{1}H NMR (600 MHz, DMSO-d_6) δ (ppm) 10.85 (s, 1H), 10.22 (s, 1H), 7.71 (d, *J* = 8.1 Hz, 1H), 7.62 (d, *J* = 8.5 Hz, 2H), 7.35 (t, 4H), 7.26 (d, *J*

= 8.1 Hz, 1H), 7.21 (s, 1H), 7.10 (dd, 6H), 7.02 (d, J = 8.5 Hz, 2H). ^{13}C NMR (151 MHz, DMSO-d_6) δ191.9, 161.6, 148.5, 147.2, 130.2, 128.4, 125.2, 124.2, 122.7, 118.0, 114.4.

2.2 Cytotoxicity assay and cell imaging

The cell and animal experiments were conducted by Professor Yang Lei's research group at Linyi University.

HeLa cells were cultured in a modified medium containing 10% fetal bovine serum. The cells were placed in a cell incubator at 37 ℃ and 5% CO_2.

Cytotoxicity assay: The cytotoxicity of the **DHBP** probe on HeLa cells was assessed using the Cell Counting Kit-8 (CCK-8) method. HeLa cells were inoculated in a 96-well plate, and different concentrations of **DHBP** probe solutions (0 μM, 2 μM, 5 μM, 8 μM, 10 μM, 20 μM, 30 μM, and 40 μM) were added to the wells, followed by a 24-hour incubation period. Subsequently, 10 μL of CCK-8 reagent was added to each well, and the plate was incubated for an additional 4 hours at 37 ℃. The absorbance was then measured at 450 nm, with each group having three replicates, and the experiment was repeated three times.

Cell culture and cell imaging: HeLa cells were cultured in Dulbecco's Modified Eagle's Medium (DMEM) containing 10% FBS and 1% antibiotics (penicillin and streptomycin) and placed in a 37 ℃ incubator with 5% CO_2. The culture medium was changed every two days to maintain the normal growth of the cells. During the experiments, the cells were incubated with the desired concentrations of probes or additives at 37 ℃ for different durations. Afterward, they were washed three times with

1 mL of PBS at room temperature and then suspended in 1 mL of PBS medium. Finally, the cells were observed under a confocal microscope.

Probe **DHBP** (20 μM) was co-incubated with HeLa cells in a cell culture incubator with a mixture of 5% CO_2 and 95% air at 37 ℃ for 5 h. The cells were then continuously irradiated with a laser for 30 min, and fluorescent pictures were taken every five minutes.

In the co-localization experiment, HeLa cells were transferred to confocal petri dishes incubated for 24 hours and then incubated with **DHBP** (20 μM) for 60 min. After washing with PBS for 3 times, commercial dyes of mitochondria were added respectively. After incubation for 30 minutes, images were recorded by confocal microscope.

Distinguishing normal and cancer cells experiment: HeLa cell and LO2 cells were incubated with probe **DHBP** (20 μM) for 60 min, respectively, and the fluorescence signals of the green channel (500−550 nm) and the red channel (651−720 nm) were collected by confocal microscopy.

In the cell inflammation experiment: HeLa cells were transferred to confocal culture dishes and cultured for 24 hours. They were then divided into two groups. One group was pre-treated with 20 μmol/L LPS for 30 minutes, while the other group was treated with 20 μmol/L LPS and apocynin. The cells were washed three times with PBS buffer. Subsequently, both groups were incubated separately with **DHBP** (20 μM) for 60 minutes, washed three times with PBS, and images were recorded using a fluorescence confocal microscope.

2.3 Establishment of mice model

In the mice inflammation experiment: healthy mice were randomly divided into three groups. One group received no treatment, another group was pre-treated with 20 μmol/L LPS for 30 minutes, and the third group was treated with 20 μmol/L LPS and apocynin. The probe was injected before imaging, and images were recorded using a fluorescence confocal microscope.

Diabetes Mouse Model: female mice used in the experiment were purchased from Beijing Wei tong li hua Experimental Animal Technology Co. Ltd. The mice were randomly divided into four groups, with three mice in each group. After an overnight fast, they were intraperitoneally injected with freshly dissolved STZ in 0.01 mol/L citrate buffer (pH = 4.5) at a dose of 150 mg/kg body weight to induce the diabetes model. The mice were then properly maintained for the following 7 days. The first group served as the control group, with no additional treatment, and the probe was injected before imaging; The second group consisted of diabetic mice, and the probe was injected before imaging; The third group consisted of diabetic mice at a dose of 80 mg/kg body weight oral metformin, and the probe was injected before imaging; The fourth group consisted of diabetic mice at a dose of 180 mg/kg body weight oral metformin, and the probe was injected before imaging; Subsequently, fluorescence imaging was performed for each group of mice.

Liver Tissue Section Experiment: liver tissues from the aforementioned experimental mouse groups were sectioned, and these tissue sections were co-incubated with the probe. Subsequently, imaging was conducted under a confocal microscope.

Organ Imaging Experiment: the major organs (liver, spleen, heart, lungs, and kidneys) of the experimental mice from the aforementioned groups were stained. These tissue sections were co-incubated with the probe and subsequently imaged under a confocal microscope.

2.4 Supplementary figures

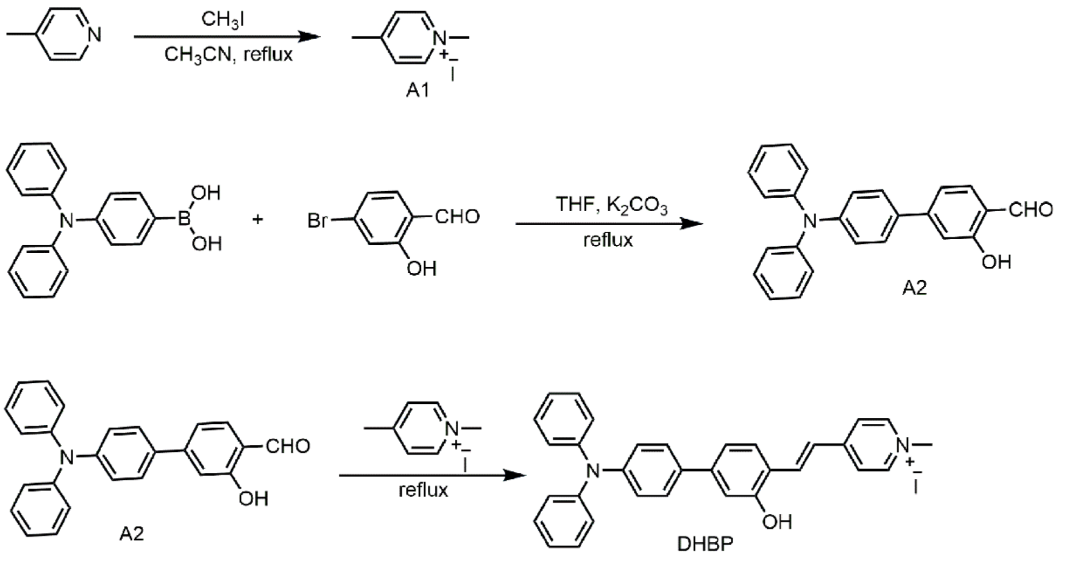

Scheme S1. Synthetic route of **DHBP**.

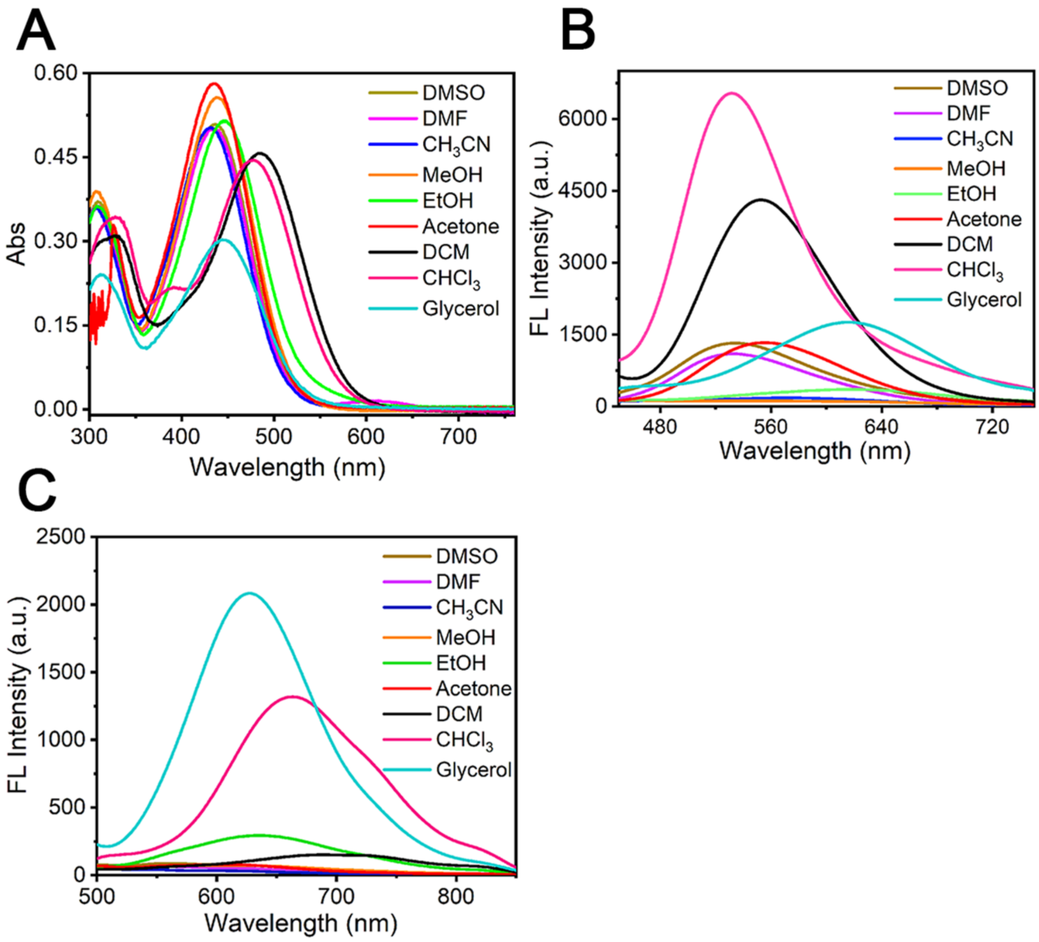

Figure S1. (A) UV-vis absorption spectra of probe **DHBP** (1.0×10^{-5} mol/L) in different solvents. Fluorescence emission spectra of probe **DHBP** (1.0×10^{-5} mol/L) in different solvents. (B, λ_{ex} = 405 nm; C, λ_{ex} = 480 nm, slits: 10 nm, 10 nm).

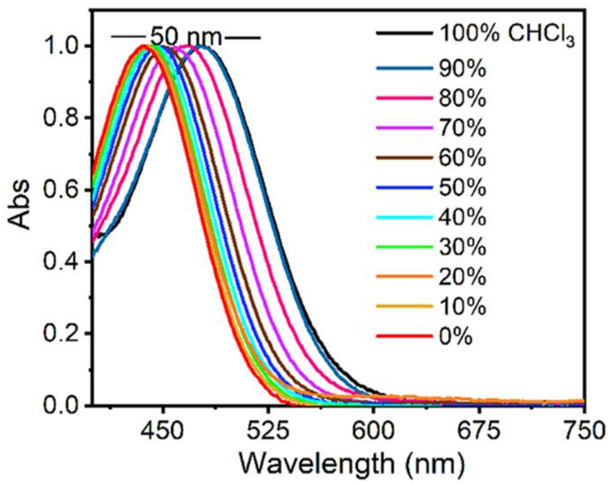

Figure S2. Normalized UV-vis absorption spectra of **DHBP** (1.0×10^{-5} mol/L) in different volumes of $CHCl_3$/DMSO solution.

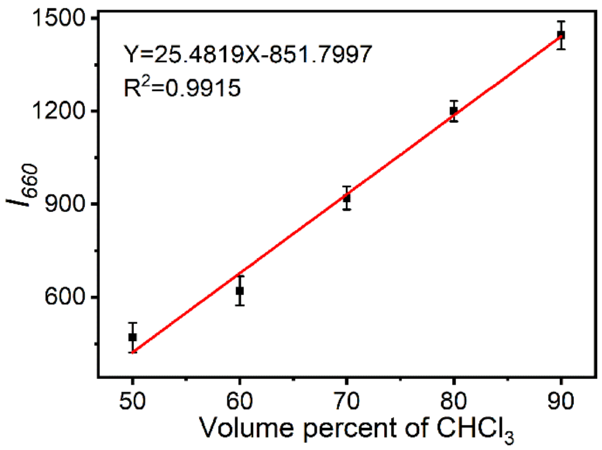

Figure S3. The linear relationship between the fluorescence intensity of probe **DHBP** at 660 nm and the percent of $CHCl_3$. (λ_{ex} = 480 nm, slits: 10 nm, 10 nm).

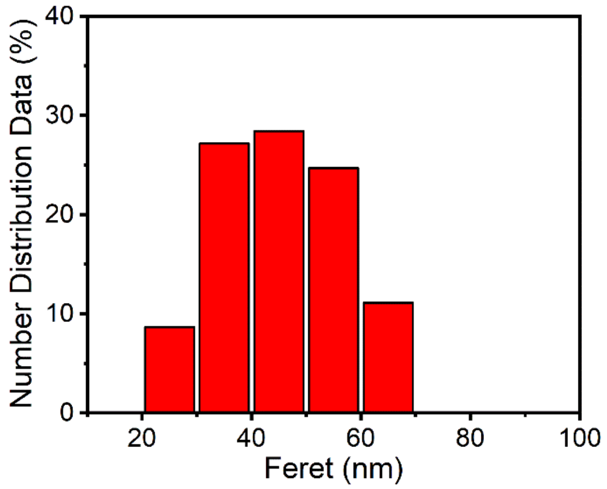

Figure S4. Particle size distribution of **DHBP** dissolved in DMSO obtained by TEM.

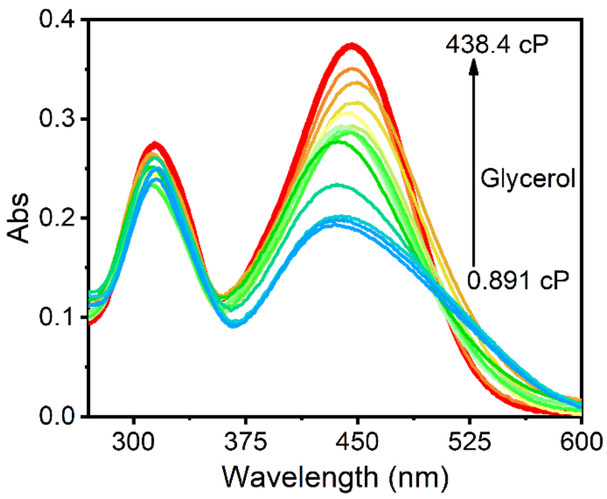

Figure S5. UV-vis absorption spectra of **DHBP** (1×10^{-5} mol/L) in different ratios of glycerol and PBS buffer solution (pH=7.3).

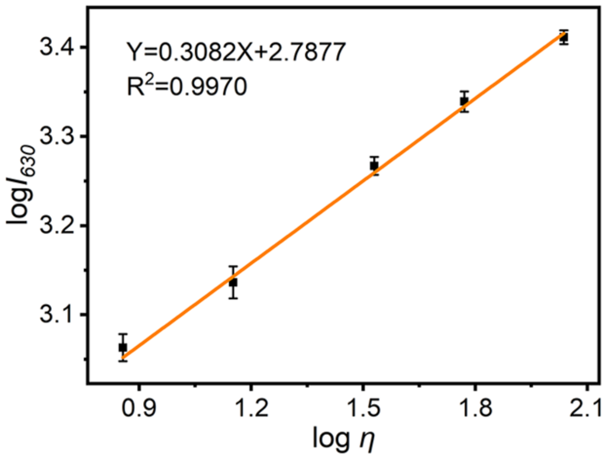

Figure S6. The linear relationship (R^2 = 0.9970) between log η of viscosity and log I_{630} of probe **DHBP** (1×10^{-5} mol/L), (λ_{ex} = 450 nm, slits: 10 nm, 10 nm).

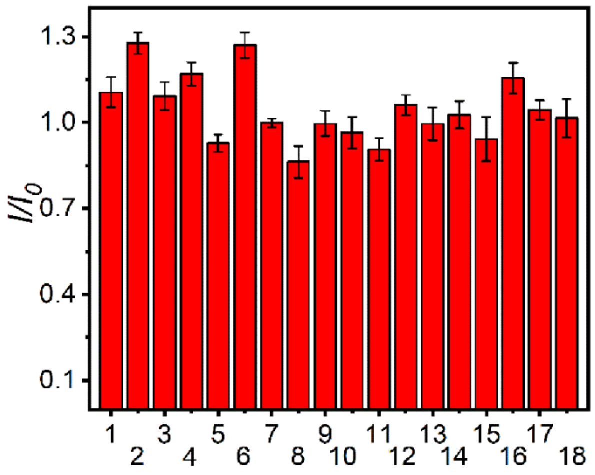

Figure S7. Fluorescence intensity ratios (I/I_0) of **DHBP** (1×10^{-5} mol/L) at 630 nm with addition of 100 μM various analytes. (1. Blank, 2. Mg^{2+}, 3. K^+, 4. Fe^{2+}, 5. Ca^{2+}, 6. Cu^{2+}, 7. Zn^{2+}, 8. Mn^{2+}, 9. Na^+, 10. F^-, 11. Br^-, 12. S^{2-}, 13. ClO^-, 14. CO_3^{2-}, 15. HSO_3^-, 16. H_2O_2, 17. Cys, 18. GSH).

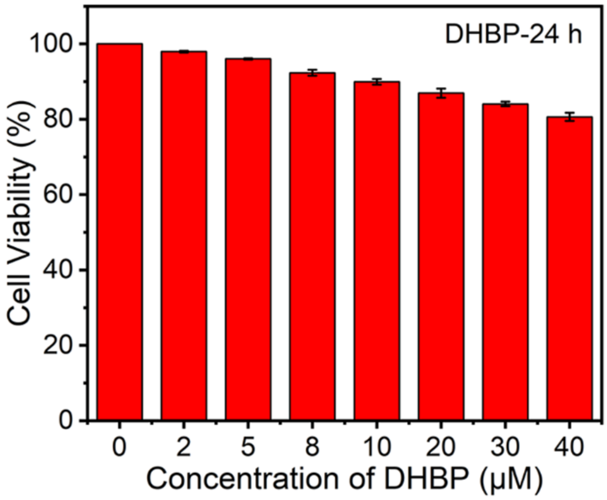

Figure S8. Cell viability after co-incubation with probe **DHBP** in HeLa cells for 24 hours.

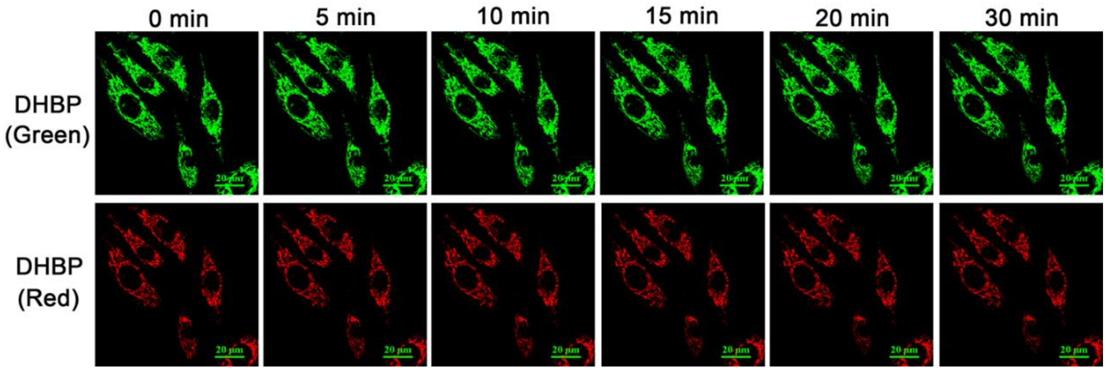

Figure S9. Fluorescence images of **DHBP** (20 μM) in HeLa cells in 30 min.

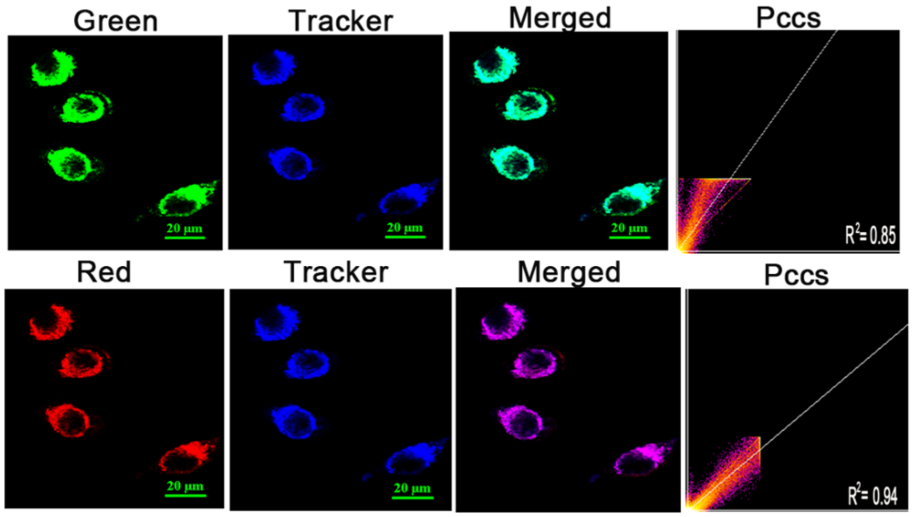

Figure S10. Colocalization effect of probe **DHBP** (20 μM) toward mitochondrial in HeLa cells.

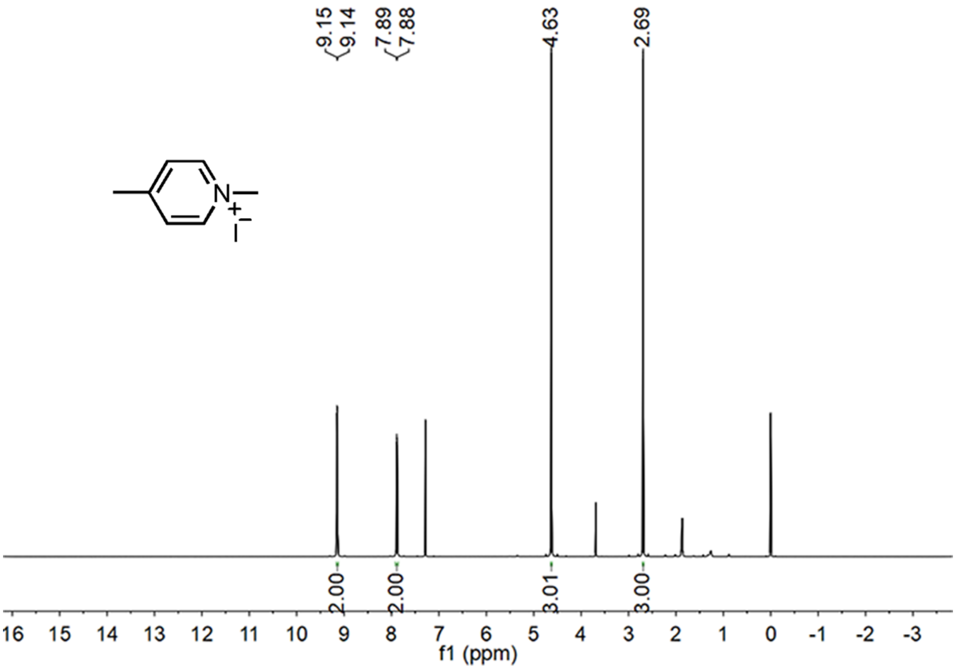

Figure S11. ^{1}H NMR spectrum of A1 (600 MHz, $CDCl_3$).

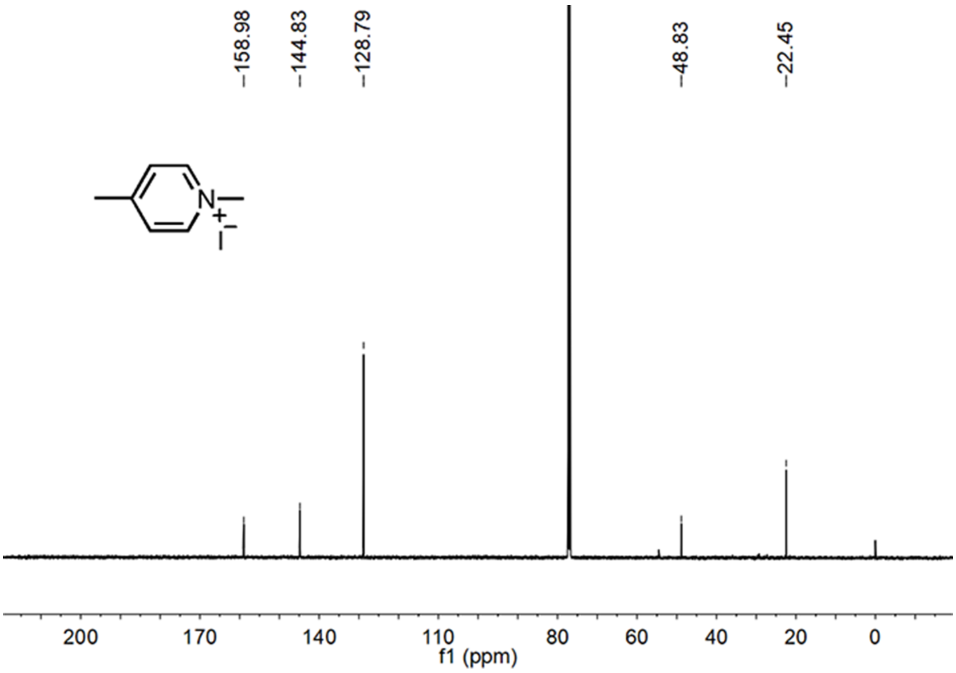

Supplement: Supplementary file 1 — im3c00128_si_001.pdf [file im3c00128_si_001.pdf]
